# Supplementary material for: Dinochromosome Heterotermini with Telosomal Anchorages
Source: Int J Mol Sci. 2024 Oct 21;25(20):11312. doi: 10.3390/ijms252011312 (PMC11508785; doi:10.3390/ijms252011312)
Supplement: Supplementary file 1 [file ijms-25-11312-s001.zip › Supplementary materials.pdf]

Supplementary materials

Article

Dinochromosome Heterotermini with Telosomal Anchorages

Alvin Chun Man Kwok †, Kosmo Ting Hin Yan †, Shaoping Wen †, Shiyong Sun, Chongping Li and Joseph Tin Yum Wong *

Division of Life Science, The Hong Kong University of Science and Technology, Clear Water Bay, Kowloon, Hong Kong SAR, China; alvink@ust.hk (A.C.M.K.); kosmo@connect.ust.hk (K.T.H.Y.); wensprussell@gmail.com (S.W.); shysun@swust.edu.cn (S.S.); cliaq@connect.ust.hk (C.L.)

* Correspondence: botin@ust.hk; Tel.: +86-852-23587343

† These authors contributed equally to this work.

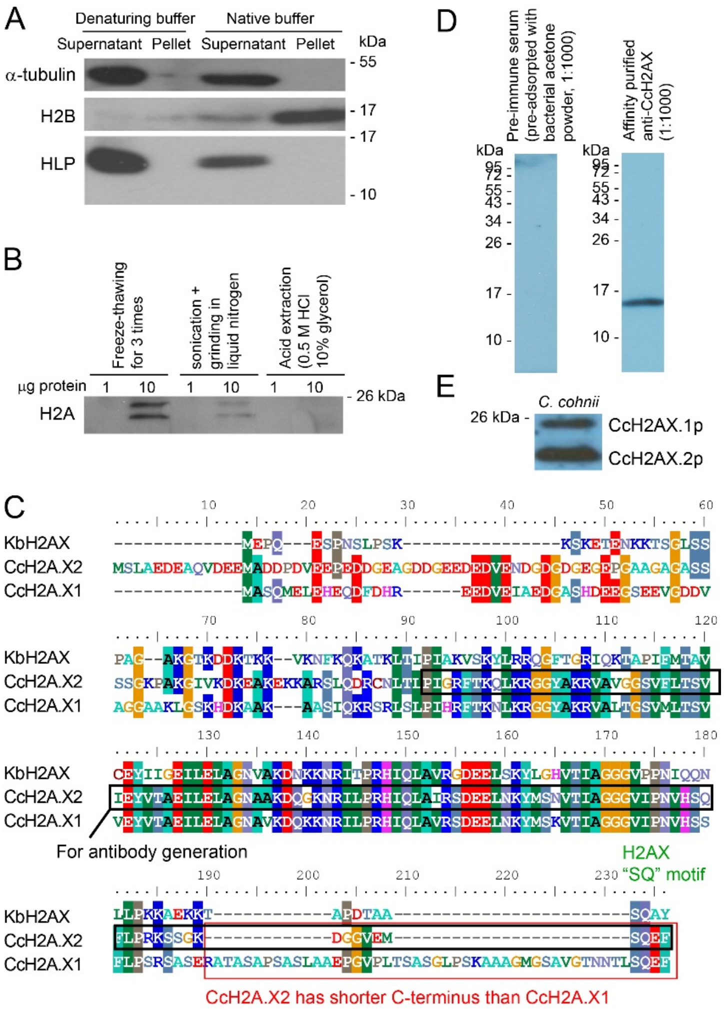

Figure S1. Antibodies against CcH2AXp and CcH2B. (A) Immunoblot analysis of extracted nuclear proteins revealed an unexpected distribution: *Crypthecodinium cohnii* histone H2B (CcH2B) was predominantly in the cell pellet, while dinoflagellate histone-like proteins (dHlps) and α-tubulin were mainly in the supernatant. (**B**)

Immunoblotting of H2A from *C. cohnii* cell lysate prepared using different cell lysis methods. Ten micrograms, but not one microgram, of total protein extract was required to produce a Western blot signal. The freeze-fracture technique was more efficient at extracting H2A protein than methods involving sonication, grinding in liquid nitrogen, and acid extraction. (**C**) Multiple sequence comparisons of histone H2A from *Crypthecodinium cohnii* and *Karenia brevis* reveal that CcH2AX2 (214 aa, pI 4.4) has a longer N-terminal but much shorter C-terminal region than CcH2AX.1 (199 aa, pI 5.75). Amino acids 93-200, covering the most conserved region of the two CcH2AXs, were used for antibody generation (black box). The two H2AX homologs would have identical mobility on SDS-PAGE. KbH2Ap had a predicted molecular weight of 17.9 kDa and shared > 40% sequence homology with CcH2A.X2p. (**D**) Antigen-purified anti-CcH2AX.2 antibodies were immunopositive to a ~14 kDa bacterial expressed truncated CcH2AX.2p (antigen) and (**E**) both CcH2AX.1p and CcH2AX.2p in *C. cohnii* cell lysate. About 50 μg of total protein extract extract was loaded per lane

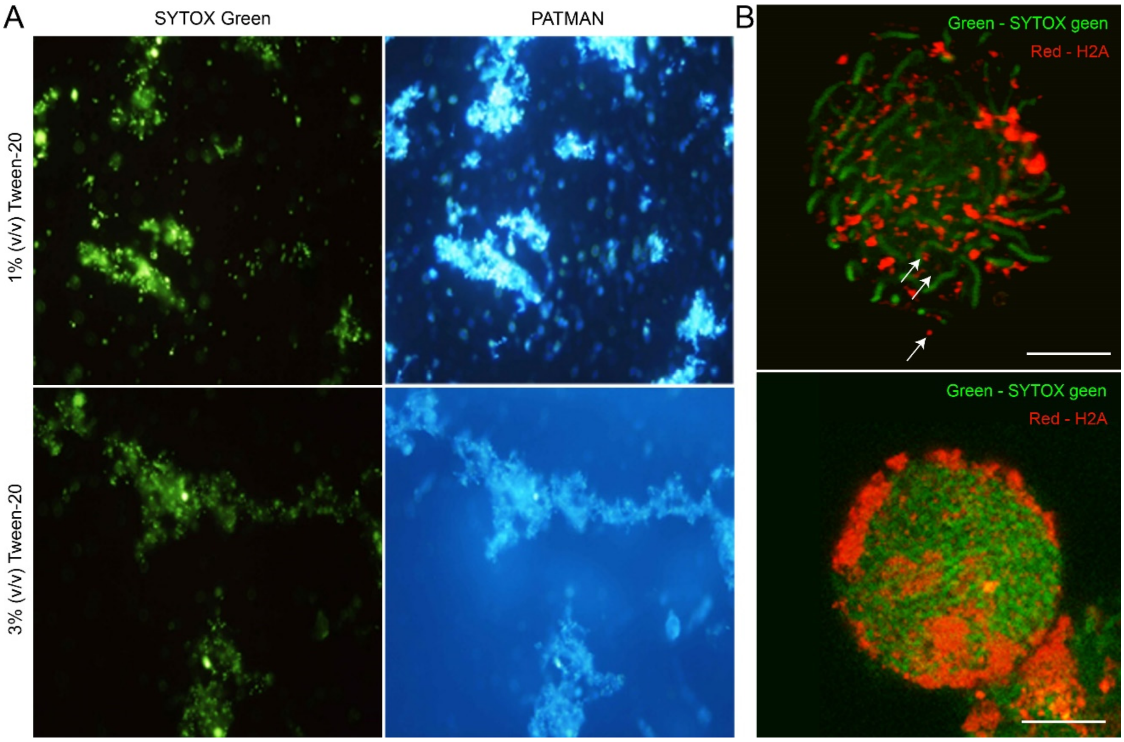

Figure S2. Anti-histone H2A immunostaining depended on chromosome decompaction. (**A**) MNase-resistant nucleic acids (stained by SYTOX Green), which were associated with the membrane (stained by PATMAN), were observed following sucrose gradient centrifugation. Tween20 at 1% and 3% concentrations were used in the preparation of the nuclear envelope. Scale bar = 10 μm. (**B**) Confocal image demonstrating specific anti-H2A immunolabeling exclusively within decompacted BFCs, forming aggregates separated from the broken nuclei but not along the pre-termini in lesser-decompacted BFCs (stage II, upper panel). Additionally, anti-H2A immunolabeling decorated the nuclear envelope (lower panel, compacted BFCs).

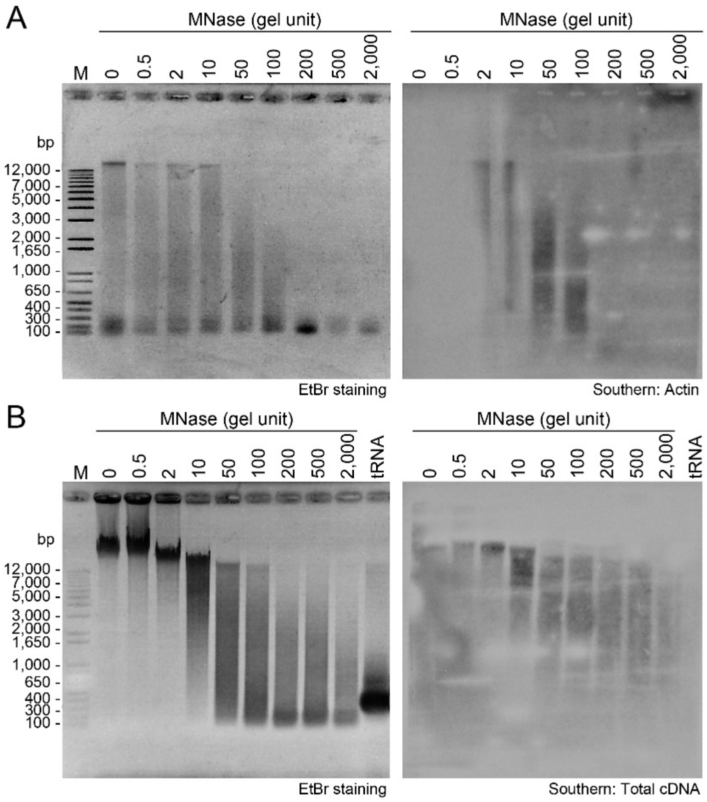

Figure S3. MNase profiling pattern with probes of gene coding regions. Southern blotting analysis of *Crypthecodinium cohnii* nuclei MNase profiling using probes specific to (**A**) actin and (**B**) total cDNA. The probes only labeled the central part of the MNase profile and did not show extended resistance as observed in the telomeric repeats.

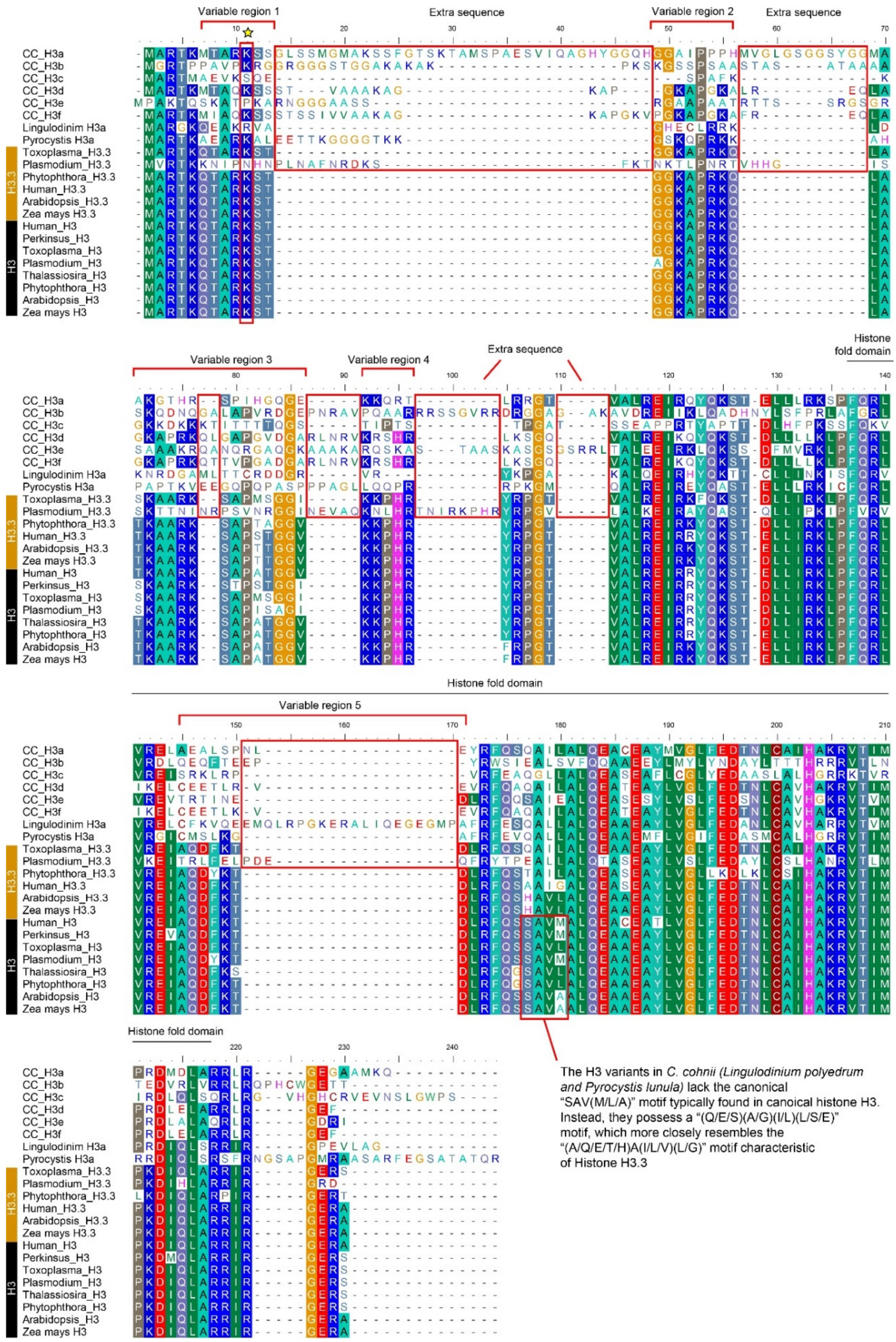

Figure S4. Comparative analysis of protein sequences and post-translational modifications among *Crypthecodinium cohnii* histone H3 variants, and the canonical human histone H3 and H3.3. Multiple sequence alignment of *C. cohnii* H3 variants

with canonical H3 proteins from other organisms, including apicomplexans, perkinsids, and diatoms (the latter two having H3 but not H3.3), revealed that *C. cohnii* H3 variants exhibit unique features distinct from both their close relatives and more distant eukaryotes (Figure S4). *C. cohnii* H3 variants show regions with highly variable sequences compared to apicomplexans and possess extra sequences in both the N-terminal and histone fold domains not found in other eukaryotes. Moreover, *C. cohnii* H3 variants do not share the "(A/Q/E/T/H)A(I/L/V)(L/G)" sequence motif conserved in many eukaryotic H3.3 proteins, nor do they possess the "SAV(M/L/A)" motif characteristic of canonical H3 in many organisms. Instead, these variants display a diverse "(Q/E/S)(A/G)(I/L)(L/S/E)" motif, which more closely resembles the H3.3 "(A/Q/E/T/H)A(I/L/V)(L/G)" motif. Despite these sequence differences, both CcH2A and CcH3 variants were still recognizable by commercially available H3 and H2A antibodies used in this study (Figures S1, S2, and Figure 6), which target the highly conserved histone fold region shared by canonical histones and histone variants. Putative methylation and acetylation site at H3K9 residue was marked with a star. Accession number: *Crypthecodinium cohnii* (H3a-f: UHA57726.1, UHA57727.1, UHA57728.1, UHA57729.1, UHA57730.1, UHA57731.1), *Lingulodinium polyedrum* (H3a [JO753891.1]), *Pyrocystis lunula* (H3a [AAN85430.1]), *Toxoplasma gondii* (H3 [XP_002365267.1], H3.3 [XP_002365267.1]), *Plasmodium falciparum* (H3 [AAO23910.1], H3.3 [KOB62391.1]), *Phytophthora infestans* (H3 [XP_002906612.1], H3.3 [XP_002904932.1]), *Homo sapiens* (H3 [CAB02546.1], H3.3 [NP_001365976.1]), *Arabidopsis thaliana* (H3 [CAB96853.1], H3.3 [CAB80666.1]), *Zea mays* (H3 [AAA33473.1], H3.3 [NP_001281232.1]), *Perkinsus marinus* (H3 [XP_002771965.1]), *Thalassiosira pseudonana* (H3 [XP_002294009.1]).

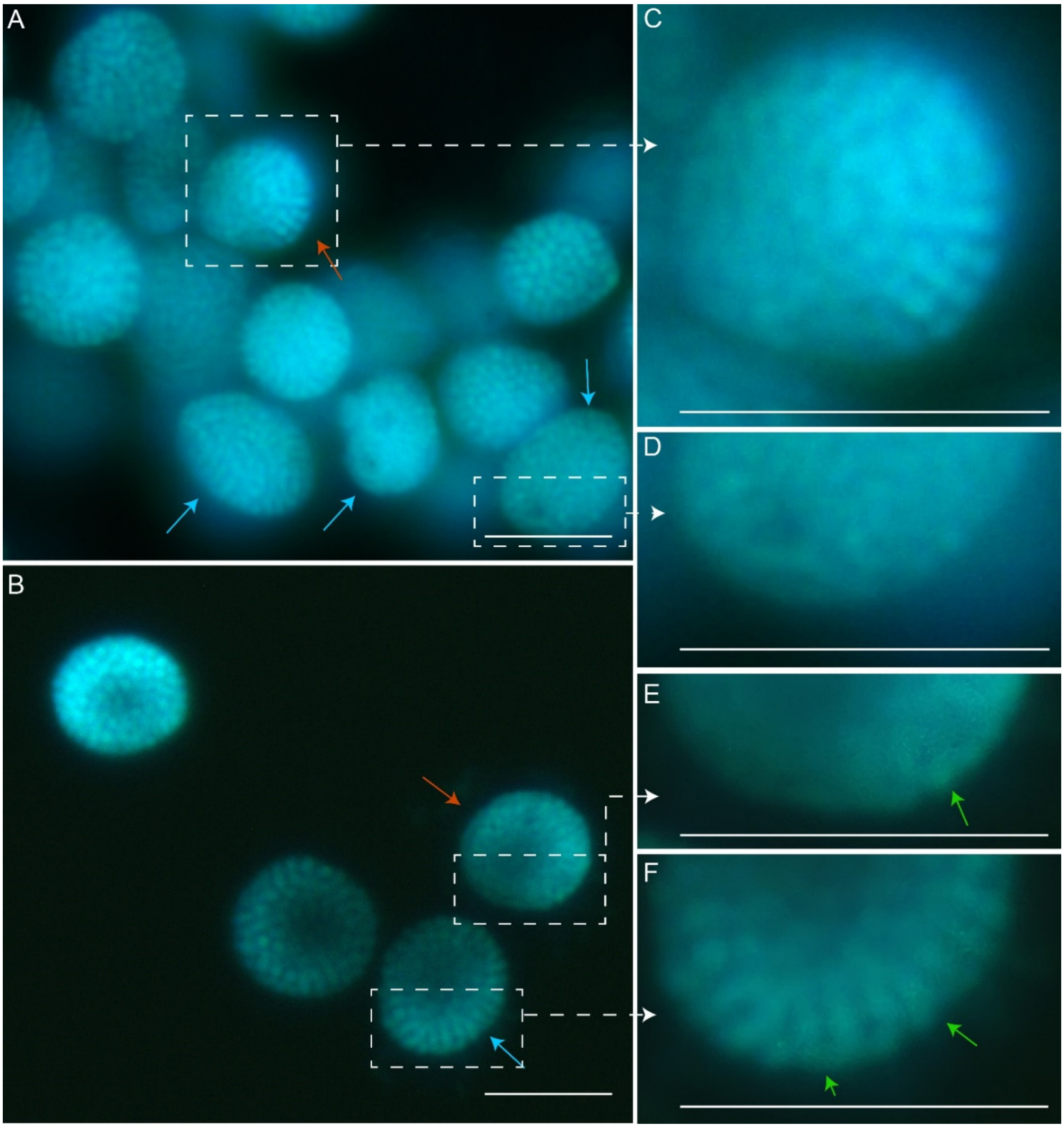

Figure S5. Comparison of untreated and psoralen-crosslinked *Karenia brevis* nuclei. Fluorescence photomicrographs of DAPI-stained, freshly isolated *K. brevis* nuclei. (**A**) Untreated nuclei. (**B**) Psoralen-crosslinked nuclei. Red arrows indicate the circular G_1 nuclei; blue arrows indicate the oval shaped G_2 nuclei. (**C, D**) Magnified views of untreated nuclei from (**A**). (**E, F**) Magnified view of psoralen-crosslinked nuclei from (**B**). Following psoralen treatment, the DAPI-stained nuclear envelope exhibited green fluorescence (green arrows). G_2 BFCs appeared more separated in psoralen-treated nuclei (**F**) compared to untreated nuclei (**D**). Scale bar = 10 μm.

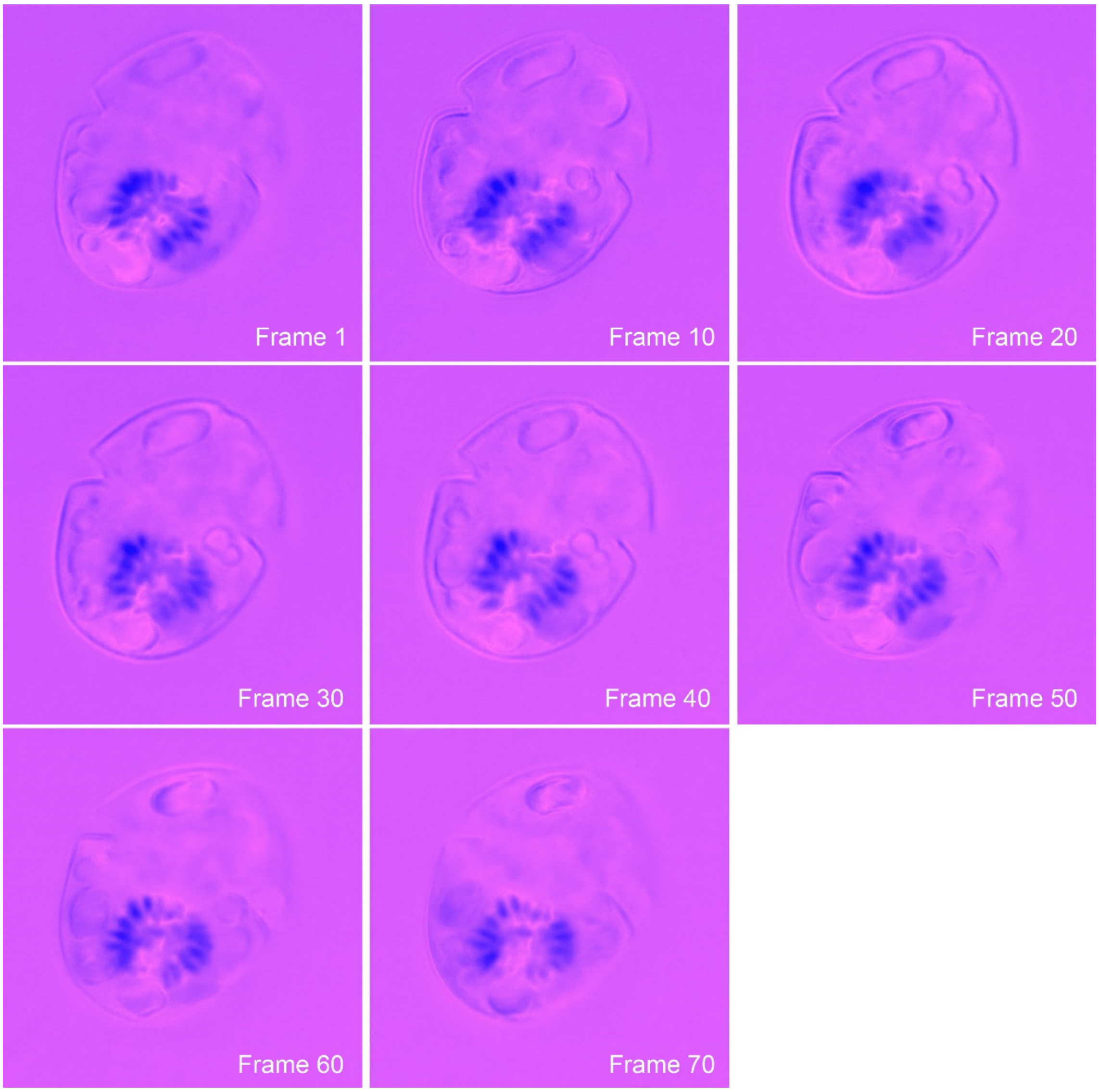

Video S1. Metripol retardance series of birefringent chromosomes in a live G_1 *Karenia* cell. A series of metripol retardance images, captured over approximately one hour, were analyzed. These frames, taken at 30-second intervals, revealed consistent relative chromosomal positions throughout the observation period.
