# Supplementary figures and images for: Dinochromosome Heterotermini with Telosomal Anchorages
Source: Int J Mol Sci. 2024 Oct 21;25(20):11312. doi: 10.3390/ijms252011312 (PMC11508785; doi:10.3390/ijms252011312)

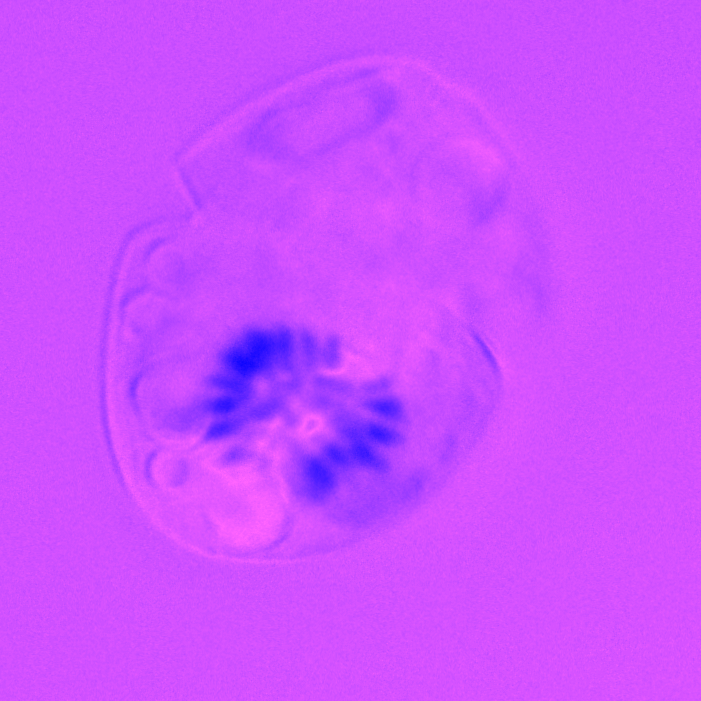

Supplement: Supplementary file 1 [file ijms-25-11312-s001.zip › Sup Ani 1.gif]
